# Influenza NG-34 T cell conserved epitope adjuvanted with CAF01 as a possible influenza vaccine candidate

**DOI:** 10.1186/s13567-020-00770-4

**Published:** 2020-04-20

**Authors:** Marta Sisteré-Oró, Gabriel K. Pedersen, Lorena Córdoba, Sergi López-Serrano, Dennis Christensen, Ayub Darji

**Affiliations:** 1 IRTA, Centre de Recerca en Sanitat Animal (CReSA, IRTA-UAB), Campus de la Universitat Autònoma de Barcelona, 08193 Bellaterra, Barcelona, Spain; 2 Virus Research and Development Laboratory, Department of Virus and Microbiological Special Diagnostics, Statens Serum Institut, Artillerivej 5, 2300 Copenhagen S, Denmark

## Abstract

Conserved epitopes are targets commonly researched to be part of universal vaccine candidates against influenza viruses (IV). These conserved epitopes need to be cross-protecting against distinct IV subtypes and to have a strong immunogenic potential. Nevertheless, subunit vaccines generally require a strong adjuvant to enhance their immunological effects. Herewith, we compare four different adjuvants differing in their immunological signatures that may enhance efficacy of a conserved hemagglutinin (HA)-epitope from IV, the NG-34, to define the most efficient combination of antigen/adjuvant to combat IV infections. Soluble NG-34 was mixed with adjuvants like aluminium hydroxide (AH) and AddaVax, known to induce Th2 and humoral responses; CAF01 which displays a biased Th1/Th17 profile and Diluvac Forte which augments the humoral response. Combinations were tested in different groups of mice which were subjected to immunological analyses. CAF01 + NG-34 induced a complete immune response with the highest IgG1, IgG2c titers and percentages of activated CD4 T cell promoting IFN-γ, IL-2 and TNF-α producing cells. Furthermore, in NG-34 stimulated mice splenocytes, cytokine levels of IFN-γ, IL-1β, IL-6, IL-10, IL-17 and TNF-α were also the highest in the CAF01 + NG-34 mouse group. This complete induced immune response covering the humoral and the cellular arms of the adaptive immunity promoted by CAF01 + NG-34 group suggests that CAF01 could be a good candidate as an adjuvant to combine with NG-34 for an efficacious vaccine against IV. However, more studies performed in IV hosts as well as studies with a challenge model are further required.

## Introduction

Purified antigens, mostly identified using in silico tools, are the constituants of subunit vaccines. These types of vaccines own numerous advantages when compared with the conventional inactivated-type vaccines, for instance: higher purity, greater safety (no need for special handling of infectious viruses) and a quicker massive production. Nevertheless, soluble antigens are often poorly immunogenic and require an adjuvant when the antigen is delivered [1]. This aspect makes the pursuit of a proper adjuvant important when developing subunit vaccines. The present study takes into account this aspect and combines an influenza virus hemagglutinin antigen (a conserved HA1-epitope NG-34) with four distinct adjuvants known to trigger different immune responses in order to determine the optimal antigen/adjuvant combination that may function effectively against IV.

Adjuvants are defined as substances whose main function is to enhance the ability of the vaccine to induce an appropriate immune response in the absence of adverse effects. Their mechanisms of adjuvanticity can be categorized accordingly to the criteria of O’Hagan and Valiante [2], which classifies them depending on their delivery system and their immunopotentiation. However, few adjuvants are currently licensed [3–5] and a long delay exists in licensing new types. This is creating inconvenience for novel emerging vaccines, mainly since they cannot utilize novel adjuvants focused on exploring novel strategies, such as generating also robust cell-mediated immune (CMI) responses [6–8].

The seasonal updated Trivalent/Quadrivalent Inactivated Vaccine (TIV/QIV) commercially available against influenza viruses (IV), covers up to 90% of human vaccines worldwide [9]. Lamentably, immune responses induced are strain-specific and do not cope with possible mutations or probable new emerging strains caused by antigenic shift. For decades, the idea of developing a universal vaccine which provides a broad-spectrum protection against a variety of IV has been pursued. In order to achieve this challenge, inducing antibodies by immunizing with conserved regions of the viral proteins has been investigated. Nonetheless, these epitope-based vaccines usually confer short-lived protection and are compromised compared to current licensed vaccines [10]. In the present experimental study, we evaluated a suitable adjuvant candidate (novel and registered) immunologically to be mixed with a well-studied HA-epitope by our group [11], the NG-34. The study pursued a prototype for a universal vaccine, thus the murine model was used in preliminary research.

NG-34 peptide, predicted by Informational Spectrum Methodology (ISM) [12, 13], is located within the site E in the N terminus of HA1 [14], in a domain close to the receptor binding site (RBS) of the HA, characterized for being relatively conserved. Its role in inducing specific antibodies as well as CD4 T cells in the pig model has been documented. Recently, it has been shown that NG-34 was effective as a pig influenza vaccine in combating against a heterologous challenge by reducing shedding and inducing neutralizing antibodies against a SwH3N2 virus [11]. In poultry, another study supported its cross-protective effect against an H7 HPAIV strain [15].

The four adjuvants tested (Alhydrogel 2.0%, AddaVax™, cationic adjuvant formulation 01 (CAF01) and Diluvac Forte^®^) were selected in view of their dissimilar immunological profiles and mechanisms of adjuvanticity (O’Hagan and Valiante [2]) summarized in Table 1. Among them, Alhydrogel 2.0% and AddaVax™ (similar to MF59^®^) are formulated in human registered vaccines while CAF01 is in clinical development stages [16]. Diluvac Forte^®^, however, is applied only in veterinary studies and has been widely utilized in swine protein vaccines [17, 18]. However, knowing that pigs are also natural influenza hosts and regarding its safety and its easy mass-vaccination delivery, we decided to include Diluvac Forte^®^ as a suitable porcine influenza adjuvant candidate.

**Table 1:** Characteristics of each of the four adjuvants employed

Adjuvant name	Adjuvant type/chemical constituents	Immunological profile	Mechanism of action (delivery system/immunopotentiation)	Stage of development	References
Alhydrogel^®^ referred as AH	Aluminum salts (Mineral salts)/Aluminum hydroxide	T_H_2, humoral	Depot effect, delayed clearance/Activate Nalp3 inflammasome	Both human and veterinary licensed vaccines	[47–51]
AddaVax™ (Invivogen); similar to MF59^®^	Oil-in-water emulsion/Squalene and polysorbate mixture	T_H_2, humoral	Enhance antigen presentation/Induce APC maturation	Licensed European seasonal influenza vaccines	[52–57]
CAF01	Particulate: Cationic liposome/Dimethyldioctadecylamonium (DDA) and α,α′-trehalose-6,6′-dibehenate (TDB)	T_H_1, T_H_17, humoral	Protect antigen from destruction/Induce APC maturation	Phase 1	[8, 19, 34, 58, 59]
Diluvac Forte™	Oil-in-water emulsion/α-tocopherol	Humoral	Enhance antigen presentation/Induce APC maturation	Approved in Animal Health	[17, 18, 60, 61]

T_H_ = T helper; Nalp3 = NACHT, LRR and PYD domains-containing protein 3; APC = antigen presenting cells.

Significant differences in the induction of humoral as well as cellular immune responses were only observed when combinations of AH + NG-34 and CAF01 + NG-34 were used. However, a combination of CAF01 + NG-34 was also effective in upregulating a wide array of cytokines as well as IgG2c antibodies, representing the most complete immune response. Nonetheless, further studies in influenza hosts are vital to evaluate the efficacy and effectiveness of the vaccine combination in natural IV infections.

## Materials and methods

### Mice handling and experimental design

Mouse experiments took place at Statens Serum Institut (SSI) animal facilities. For this adjuvant testing research study, a total of twenty-seven 7–8 week old female mice of the inbred strain C57BL/6 (Envigo, Huntingdon, UK) were employed. Upon arrival, all mice were allowed to rest for 1 week previous to the first immunization. Moreover, they were randomly distributed into six separate cages according to the number of groups required in the experiment (groups A to F). Animals were provided food and water ad libitum.

Six groups (group A to F) of *n* = 5 (in exception of group A, *n* = 3 and group B, *n* = 4) were immunized twice subcutaneously (s.c) at the base of the tail with a 21-day intervals. In group A, mice were not immunized (control group). In the rest of the groups (groups B to F), a dose of 15 µg/animal of conserved HA-epitope NG-34 was injected alone (group B) or adjuvanted with AH (group C), AddaVax (group D), CAF01 (group E) or Diluvac Forte (group F) (Table 2). Three weeks after the second immunization, all animals were euthanized.

**Table 2:** Representation of the six mice groups (Groups A-F)

Group	*n*	Antigen	Adjuvant	Inoculation route
A	3	–	–	–
B	4	NG-34	–	s.c.
C	5	NG-34	Alhydrogel 2.0%	s.c.
D	5	NG-34	AddaVax™	s.c.
E	5	NG-34	CAF01	s.c.
F	5	NG-34	Diluvac Forte^®^	s.c.

*n*= number of mice; s.c = subcutaneously.

Sampling from each individual of the study was performed 3 weeks after the second immunization, at day 42 (termination day) when blood, spleens and inguinal lymph nodes were collected.

### Adjuvants and vaccine preparation

CAF01, Diluvac Forte^®^ (Statens Serum Institut, Copenhagen, Denmark), AddaVax™ (Invivogen, Toulouse, France) and aluminium hydroxide (Al(OH)3, Alhydrogel 2.0%) (Croda Biosector, Frederikssund, Denmark) were the adjuvants employed.

Mouse vaccines with an end volume of 200 µL were prepared by mixing the antigen (15 µg/animal/dose) dissolved in Tris-buffer with 9% (w/v) trehalose, in a ratio of 1:1 with the adjuvant except for Alhydrogel 2.0% (each vaccine comprised 500 µg aluminum content/200 µL) [19, 20] and group A which was not immunized.

CAF01 was freshly prepared in agreement with other studies and comprised: 250 µg/50 µg dimethyldioctadecylammonium (DDA)/trehalose 6,6 V-dibehenate (TDB)/100 µL [8, 19].

### Cells, antigens and viruses

Madin-Darby Canine Kidney (MDCK, ATCC CCL-34) cells were used in the seroneutralization assays. They were properly cultured in Dulbecco's Modified Eagle Medium (DMEM) supplemented with 10% fetal bovine serum (FBS), 1% penicillin/streptomycin and 1% l-glutamine.

NG-34 epitope predicted by ISM was synthesized by GL Biochem Ltd, Shanghai, China. Its sequence corresponds with 34 amino acids in the HA1 region from the strain A/Catalonia/63/2009 (pH1N1) [GenBank ACS36215] [11].

Purified HA from A/California/04/09(H1N1)pdm09 and from A/Aichi/2/1968(H3N2) were purchased from Sino Biological, Beijing, China (cat. no. 40340-V08B and 11707-V08H, respectively).

SwH1N1 (A/swine/Spain/003/2010 H1N1 IV) [GenBank JQ319725 and JQ319727] and SwH3N2 (A/swine/Spain/003/2010 H3N2 IV) [GenBank JQ319724 and JQ319726] were the viruses used.

### Organ preparation

Spleens and lymph nodes were treated as follows: they were forced through a 70  μM filter nylon mesh (BD Biosciences, San Jose, CA, USA), suspended in Roswell Park Memorial Institute (RPMI) media without FBS and further centrifuged for 5 min at 1800 rpm. At that point, cells were resuspended in 1 mL using complete RPMI (cRPMI) (supplemented with HEPES, penicillin–streptomycin, sodium pyruvate, l-glutamine and non-essential amino acids) with 10% FBS (v/v). Finally, all samples were counted using NucleoCassetes (ChemoMetec A/S, AllerØd, Denmark) on the NucleoCounter^®^ NC-100™ (reagents and materials from ChemoMetec A/S, AllerØd, Denmark).

### Immunoassays in mice for antigen-specific serum antibodies

Maxisorb Plates (Nunc) were coated with 2 µg/well of either NG-34 epitope or HA: A/California/04/09 (H1) in buffer carbonate bicarbonate pH = 9.6 and left to incubate overnight at 4 °C. The next day, the plates were blocked during 1 h 30 min at room temperature (RT) using 2% BSA PBS solution. Mouse sera were analyzed in threefold dilution series in PBS with 1% BSA, starting with a 30-fold dilution. After a 2-hour incubating period at RT and three washes with 0.2%Tween20 PBS buffer, HRP-conjugated secondary antibodies (rabbit anti-mouse IgG1; Southern Biotech, Birmingham, AL, USA; and IgG2c; Invitrogen, San Diego, CA, USA) were added with 1%BSA PBS at dilutions: 1:20 000 (IgG1) or 1:5000 (IgG2c) and incubated during 1 h at RT. Reiteratively, the plates were washed and developed using 3, 3′, 5, 5′-tetramethylbenzidine (TMB) substrate (Kem-En-Tec Diagnostics, Kuldyssen, Denmark). The reaction was later stopped by adding 0.2 M H_2_SO_4_ and the plates were read using a two-step fully automated ELISA (Hamiltion Starlet System, Switzerland) at 450–620 nm wavelength.

### Intra-cellular flow cytometry staining for mice splenocytes stimulated in vitro with NG-34 antigen

In splenocytes, the production of cytokines IFN-γ, TNF-α, IL-2 and IL-17 was assessed in the T cell population. For that purpose, cells were stained similarly as described in [21] but with few modifications. Briefly, splenocytes (10^6^ cells/well) were stimulated with 2 µg/mL of NG-34 peptide during 1 h at 37 °C and subsequently, incubated 6 h at 37 °C with brefeldin A (2.5 µg/mL) in order to block cytokine production. After this period, cells remained O/N at 4 °C. Additionally, from each group two pools were differentially made: not stimulated (negative control) or stimulated with PMA/ionomycin (positive control). Next day, all cells were stained utilizing the antibodies: CD4-BV786 (BD Pharmingen, San Diego, CA, USA; 56331), CD44-FITC (eBioscience, Frankfurt, Germany; 11-0441), CD8-PerCpCy5.5 (eBioscience, 45-0081). The cells were then permeabilized using fix/perm (BD) and washed in permeabilization wash (BD). Subsequently, the cells were stained with IL-2-APC-Cy7 (BD Pharmingen, 560547), TNF-α-PE (eBioscience, 12-7321), IFN-γ-PE-Cy7 (eBioscience, 25-7311) and IL-17a-APC (eBioscience, 17-7177). The cells were acquired on a FACS Fortessa instrument using DIVA software (BD Bioscience, USA) and all the data analyzed using FlowJo software (Tree Star Inc.). Upon their acquisition, the cells were gated following this pattern: live > singlets > lymphocytes > CD3 + > CD4 + versus CD8 + . Furthermore, cytokine-producing cells (IFN-γ, IL-2, IL-17 and TNF-α) within the CD4 + CD44 high population were measured. Compensation beads (BD Pharmingen, USA) were used to compensate the fluorophores.

### Flow cytometry staining in mice for cells in the inguinal lymph nodes

Cells from the inguinal lymph nodes were collected from all individuals; the cells were plated (10^6^ cells/well) and treated with Mouse BD Fc Block™ (BD Pharmingen, 553142) to block non-antigen-specific bindings of immunogloblulins to Fcγ III and Fcγ II receptors. Two panels of antibodies were used to stain cell populations in the germinal centers (GC) of the lymph nodes; GC B cells (B220^+^ IgD^−^ CD38^−^ GL7^+^) and T follicular helper cells (TFH) (B220^−^ CD4^+^ PD-1^+^ CXCR5^+^). The following antibodies were mixed in PBS 1% FBS: GL7-FITC (BioLegend GmbH, Koblenz, Germany; 144604), IgG1-PE (BD Pharmingen, 550083), B220-PerCP-Cy5.5 (BD Pharmingen, 552771), CD38-PE-CY7 (eBioscience, 25-0381) and IgD-BV786 (BD Pharmingen, 563618) to stain the B cells in the GC. To stain the TFH cell population, the following antibody panel was used: CD4-FITC (BD Pharmingen, 553047); CD279(PD1)-PE (BD Pharmingen, 551892); CD45R(B220)-PerCP-Cy5.5 (eBioscience, 65-0865-14), CXCR5-BV421 (BD Pharmingen, 562889) and live/dead-EF780 (BD Pharmingen, 562889). After an incubation period of 30 min at 4 °C, the cells were acquired on the FACS LSRII instrument using DIVA software (BD Bioscience, USA) and all the acquired data analyzed using FlowJo software (Tree Star Inc.). Compensation beads (BD Pharmingen, USA) were used to compensate the fluorophores.

### Mouse cytokine assay

Splenocytes were seeded (2 × 10^5^/well) and stimulated with the NG-34 epitope (5 µg/mL); media cRPMI (as negative control) and concavalin A (1 µg/mL) (as positive control) (GE Healthcare, Marlborough, MA, USA). The supernatants were harvested after 72 h incubation at 37 °C and a 9-plex panel MSD standard Th1/Th2/Th17 which checks IFN-γ, TNF-α, IL- 5, IL-10, IL-13 and IL-17 cytokines together with two U-plex panels for IL-6 and IL-1β detection were ran following the manufacturer’s instructions (MSD, Rockville, MD, USA).

### Hemagglutination inhibition (HI) assay and serum neutralization test (SNT)

Specific hemagglutination inhibition (HI) titers in mice sera were determined as the reciprocal of the later dilution of sera that inhibited hemagglutination. The protocol was adjusted to the recommendations provided by the World Organization for Animal Health (OIE) [22]. Samples from each group were pooled and ran in duplicates. A positive and negative reference serum (GD Animal Health, Deventer, The Netherlands) were also included in the assays to validate the technique.

Specific seroneutralizing antibody titers were evaluated. First, samples from each mice group were pooled, heat inactivated (56 °C for 30 min), diluted two-fold with DMEM (supplemented with 1% penicillin/streptomycin and 1% l-glutamine) and incubated for 2 h at 37 °C with 100 TCID_50_/well with the SwH1N1 (A/swine/Spain/003/2010 H1N1 IV) and SwH3N2 (A/swine/Spain/003/2010 H3N2 IV) previously treated with porcine-trypsin (Sigma-Aldrich, St. Louis, MO, USA). Later on, the mixture of sera-virus was transferred into pre-washed plates with confluent MDCK cells and kept at 37 °C with 5% CO_2_. After 7 days, the cytopathic effect (CPE) was read and the titers were expressed as the reciprocal dilution of serum that neutralized 100 TCID_50_ of the challenged strain in 50% of the cultured replicates. Furthermore, in each plate there were added media controls (no virus), virus controls (no serum) and positive and negative reference sera from GD Animal Health, The Netherlands.

### Statistical analysis

For statistical analysis, Prism 6 software (GraphPad v6.01, San Diego, CA, USA) was used. One-way ANOVA or a non-parametric test (Friedman test) followed by Dunnett multiple comparisons test was performed (assigning as control group: group B, the non-adjuvanted group). Statistically significant differences detected are illustrated by asterisks in each figure and explained in the corresponding figure legends.

## Results

### Specific humoral immune responses after 2nd vaccination

**Figure 1:**
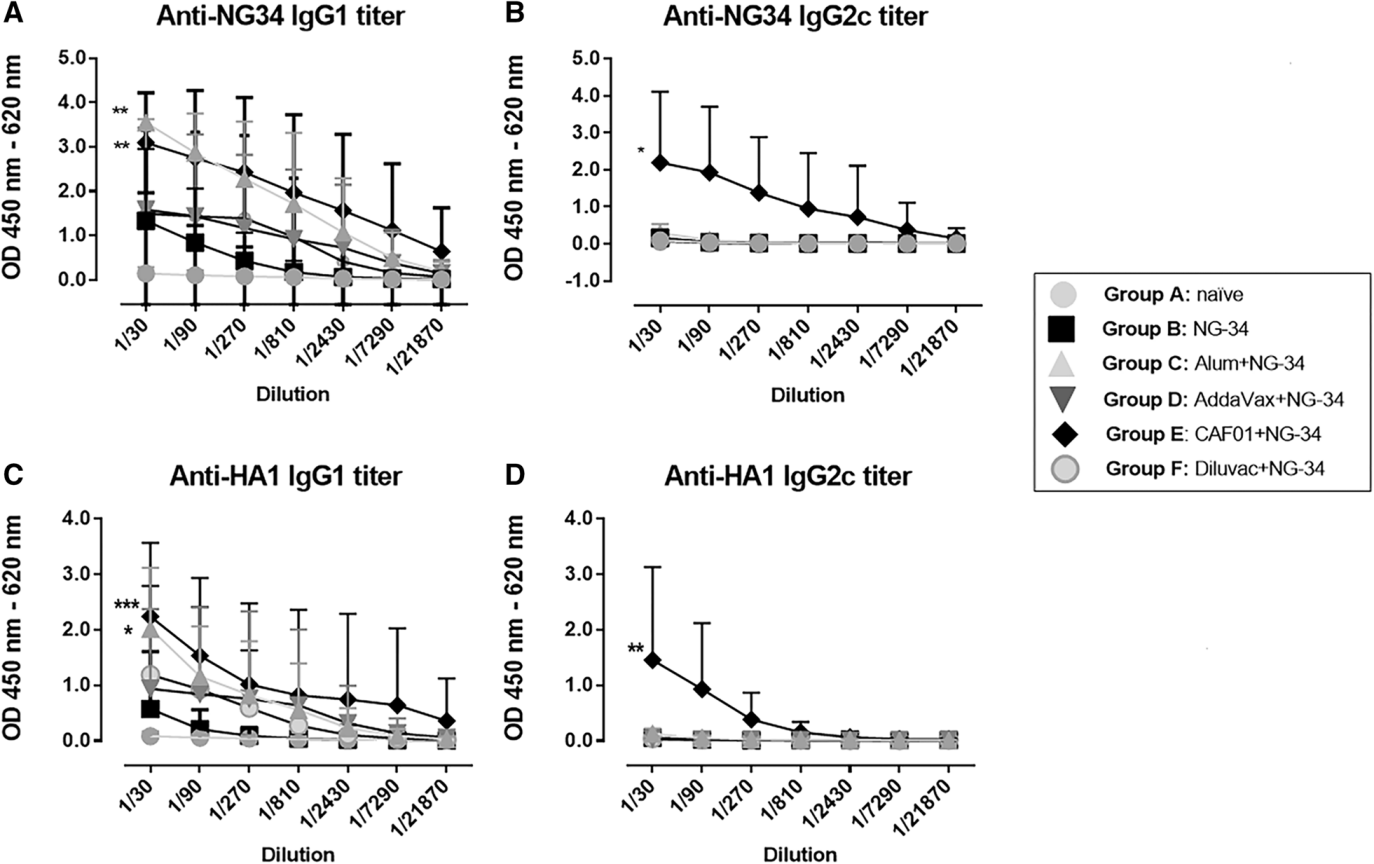
**Specific humoral responses elicited after immunizations with different adjuvant-NG34 combinations**. Serum samples from individual mice from groups A–F were collected after second immunization and analyzed in serial dilutions for IgG1 and IgG2c titers. **A** NG34-specfic IgG1 and **B** IgG2c antibody titers and HA1-specific **C** IgG1 and **D** IgG2c antibody titers. Adjuvanted groups were compared to group B (the non-adjuvanted group) using a non-parametric test (Friedman test) followed by Dunnett’s post-test. *P < 0.05; **P < 0.01, *** *P* < 0.001, ****P < 0.0001.

IgG1 and IgG2c antibody responses were analyzed against the NG-34 epitope and against the complete HA from A/California/04/09 (H1N1) (Figure 1). NG34-specific IgG1 titers were higher in AH + NG-34 and CAF01 + NG-34 groups, displaying statistically significant differences (*P* < 0.05) when compared with the non-adjuvanted group (Figure 1A). Regarding NG34-specific IgG2 titers, the CAF01 + NG-34 mice group achieved the highest titers with statistically major significant differences (*P* < 0.0001) when compared to the non-adjuvanted group (Figure 1B). The rest of the adjuvanted groups (AH + NG-34, Addavax + NG-34, Diluvac Forte + NG34) were barely inducing IgG2c titers. Furthermore, HA1-specfic IgG1 and IgG2c titers were also in concordance with the NG-34 specific antibody titers (Figures 1C and D). The AH + NG-34 group elicited high HA1-specific antibody levels (*P* < 0.05) but only the CAF01 + NG34 group achieved significant statistical differences for both of the IgG1 (*P* < 0.001) and IgG2 (*P* < 0.01) antibody titers against the non-adjuvanted group (*P* < 0.01).

### Specific cellular immune responses in the spleen

**Figure 2:**
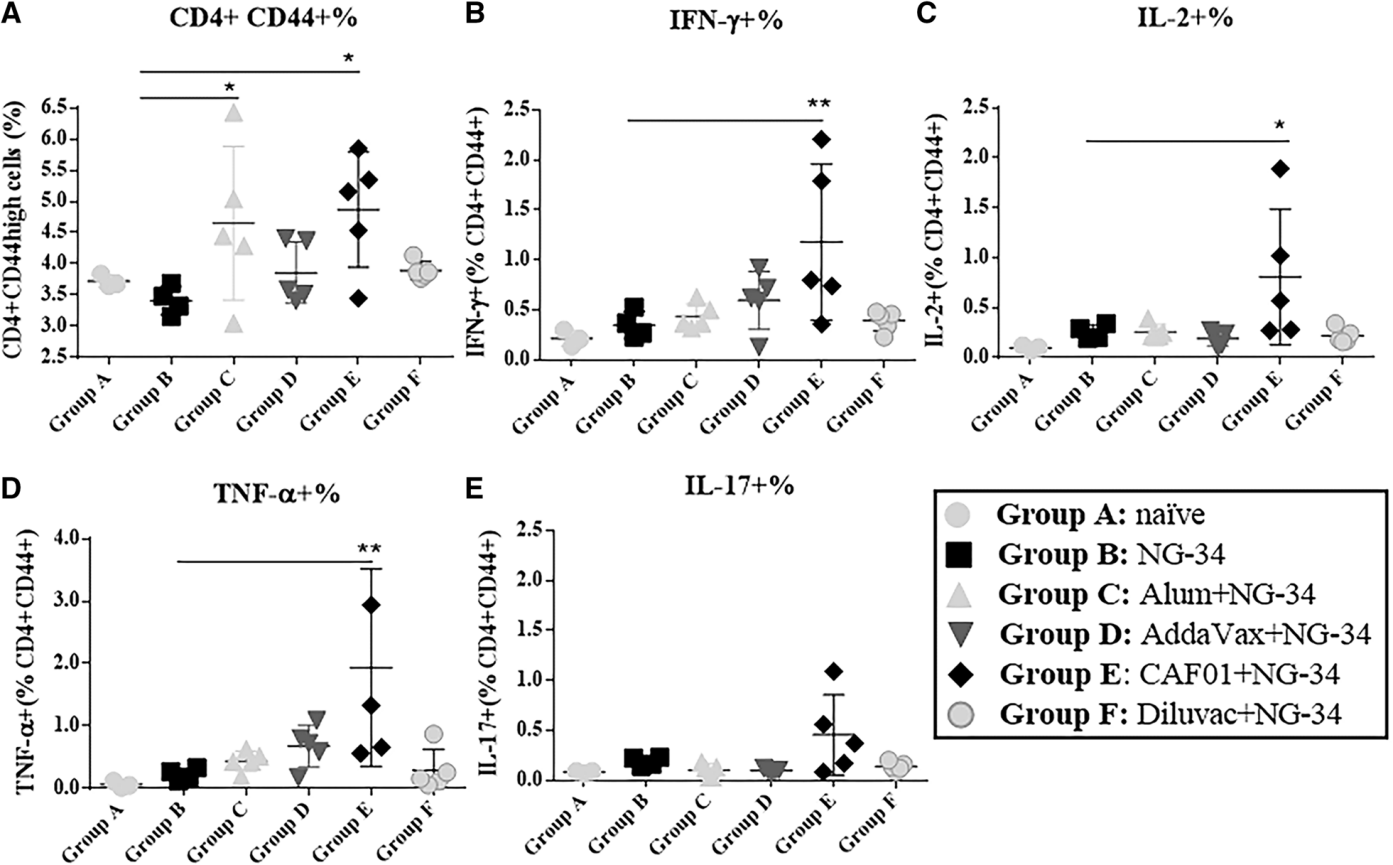
**Cellular responses in the spleens of immunized mice**. NG34-stimulated spleen cells from individual mice (groups A–F) were collected and analyzed for cytokine production by flow cytometry. Percentages of **A** CD4 + CD44high, **B** CD4 + CD44high IFN-γ, **C** CD4 + CD44high IL-2, **D** CD4 + CD44high TNF-α and **E** CD4 + CD44high IL-17 (2) are represented.

NG-34 antigen-specific cellular response was measured in the spleen. Intracellular CD4 and/or CD8 specific cytokines produced upon NG-34 stimulation was analyzed by flow cytometry. While in all the groups CD4 T cell response was observed, the CD8 T cell response was merely present. In parallel, percentages of CD4 + CD44high T cells in NG34-stimulated spleens were also determined. The AH + NG-34 and CAF01 + NG-34 groups showed the highest percentages (*P* < 0.05) in comparison to group B (Figure 2A). Only the CAF01 + NG-34 mice group demonstrated significant differences in intracellular cytokine, IFN-γ (*P* < 0.001), IL-2 (*P* < 0.05) and TNF-α (*P* < 0.01) produced by NG-34 stimulated spleens (Figures 2B–D). Additionally, IL-17 producing cells were the highest in the CAF01 + NG-34 group although no statistical differences were observed among different adjuvant/NG-34 combinations (Figure 2E).

**Figure 3:**
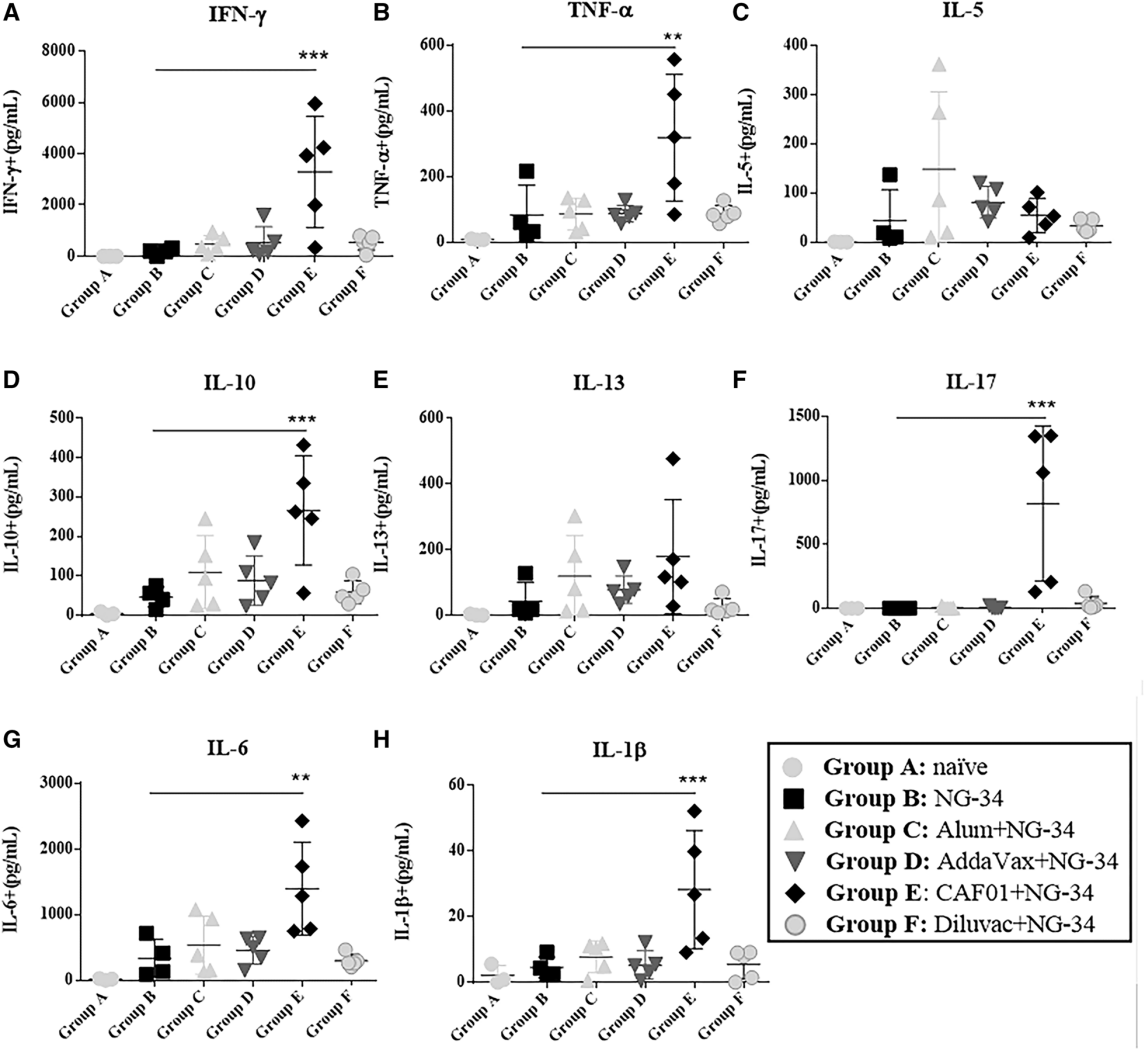
**Cellular responses in the supernatants of NG-34 stimulated spleens of immunized mice**. Harvested supernatants of NG34 stimulated spleens from individual mice from groups A-F were collected to determine the levels of **A** IFN-γ, **B** TNF-α, **C** IL-5, **D** IL-10, **E** IL-13, **F** IL-17, **G** IL-6 and **H** IL-1β. Adjuvanted groups were compared to group B (the non-adjuvanted group) using Ordinary One-way ANOVA test followed by Dunnett’s post-test. *P < 0.05; **P < 0.01, ***P < 0.001, ****P < 0.0001.

Supernatants of NG34-stimulated spleens harvested after 72 h were analyzed with a multiplex system for different cytokines like IFN-γ, TNF-α, IL-5, IL-10, IL-13 and IL-17 (Th1/Th2/Th17 type). Similarly, IL-6 and IL-1β levels were also evaluated (Figure 3). Statistically significant differences in IFN-γ (*P* < 0.001), TNF-α (*P* < 0.01), IL-10 (*P* < 0.001), IL-17 (*P* < 0.001), IL-6 (*P* < 0.01) and IL-1β (*P* < 0.001) cytokine release were only detected in the CAF01 + NG-34 group when compared to the non-adjuvanted group (Figures 3A, B, D, F–H). IL-5 in the AH + NG-34 group (Figure 3C) and IL-13 in the CAF01 + NG-34 group (Figure 3E) were detected at low concentrations although no statistically significant differences were observed in IL-5 and IL-13 among the groups.

### Alteration in the inguinal lymph node cell population in immunized mice

**Figure 4:**
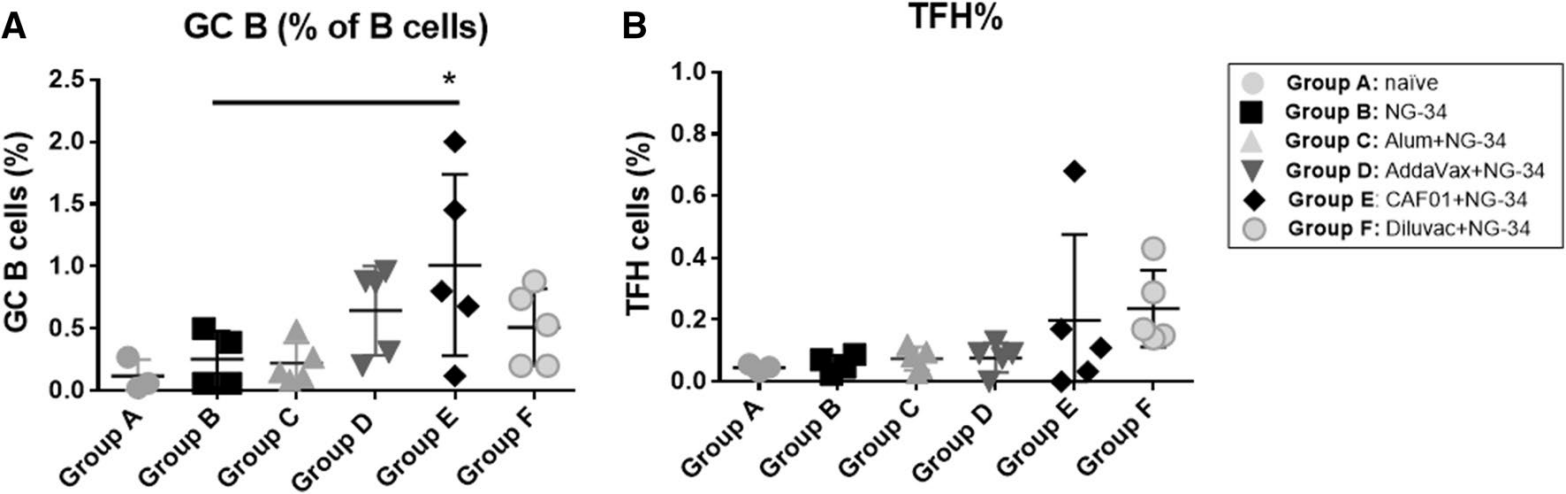
**Changes in inguinal lymph node population**. Lymph nodes samples from individual mice (groups A-F) were collected and analyzed by flow cytometry **A** the percentage of **B** cells in the GC (**B**) the percentage of TFH. Adjuvanted groups were compared to the non-adjuvanted group using Ordinary One-way ANOVA test followed by Dunnett’s post-test. *P < 0.05; **P < 0.01, ***P < 0.001, ****P < 0.0001.

Differences in the number of cells present in the GC of lymph nodes were detected. Significantly higher percentages of B cells (*P* < 0.005) were observed in the CAF01 + NG-34 group (Figure 4A). In contrast, no statistical differences were observed in TFH cells, although in CAF01 + NG34 and Diluvac Forte + NG-34 groups an increase in TFH was noticed (Figure 4B).

### Obtained HI and SNT titers in mouse sera

HI titers were negative in all mice sera for each of the tested IV. SNT titers were either negative or low (1:10) in CAF01 + NG-34 and Diluvac Forte + NG-34 groups against SwH1N1 virus. Against SwH3N2 virus, both groups (CAF01 + NG-34 and Diluvac Forte + NG-34) achieved a 1:40 titer (Table 3).

**Table 3:** SNT titers obtained from the six mice groups (groups A-F) against SwH1N1 and SwH3N2 isolates

Group	Adjuvant	SNT titers	
		Against SwH1N1	Against SwH3N2
A	–	–	–
B	–	–	–
C	Alhydrogel 2.0%	–	–
D	AddaVax™	–	–
E	CAF01	1:10	1:10
F	Diluvac Forte^®^	1:40	1:40

SNT = serum neutralization test.

## Discussion

Many immunogens/subunit antigens do not show strong immunogenicity, making the use of adjuvants necessary to reinforce their immune effect. In the present study, we focused on selecting the most suitable adjuvant that could better aid immunogenically when administered in combination with the NG-34 conserved HA-epitope.

Various groups of mice were either not immunized or immunized with NG-34 epitope alone or combined with adjuvants: AH, AddaVax, CAF01 and Diluvac Forte^®^. Interestingly, mice vaccinated with CAF01 + NG-34 elicited specific humoral and cellular immune responses to the conserved NG-34 HA-epitope (Figures 1 and 2), a dual immune induction widely considered ideal for an IV vaccine.

The AH + NG-34 group induced a polarized Th2-type response leading to significantly higher NG34-specific IgG1 titers (Figures 1A and 2A), and CAF01 combined with NG-34, promoted the highest levels of IgG1 (Figure 1A). The CAF01-NG-34 group also induced an IgG2c response, being the only peptide/adjuvant combination capable of inducing such a response (Figure 1B) which is in line with other studies published using other antigens and CAF01 [8, 20]. These induced IgG2a/c titers are regarded as an important component for IV vaccines since they mediate protection when virus neutralizing titers are absent [23, 24].

Strong HI titers were not induced by any of the evaluated epitope/adjuvant combinations. Only CAF01 + NG-34 and Diluvac Forte + NG-34 groups displayed SNT titers albeit at low levels (Table 3). These results did not correlate with the high HI titers induced when using MF59 adjuvant in IV vaccines [25, 26]. Perhaps, MF59-like adjuvants (Addavax) function better when the complete HA or IV strain are included in the vaccine formulation [27, 28]. Furthermore, the Addavax + NG-34 group did not boost Th1 and Th2 as demonstrated in other studies [25, 26]. In contrast, our results were concordant with results using CAF01 combined with TIV, which conferred protection in an IHA-independent manner [29].

To achieve cross-protection between heterologous strains, an appropriate T-cell response seems to be necessary [30]. In this work, the highest cellular responses were exhibited by the AH + NG-34 and CAF01 + NG-34 groups (Figure 2A). The results obtained in the AH + NG-34 partly correlated with the trend for a predominant Th2 differentiation and Th2-type cytokine profile, usually promoted by aluminum salts [31, 32]. Statistically significant increase in CD4 CD44high population and highest IFN- γ, IL-2 and TNF-α responses (Th1-type cytokines) were observed in mice from CAF01 + NG34 (Figures 2A and C) as also reported in other studies [6, 19, 33, 34]. Significantly higher titers of IFN-γ, TNF-α, IL-10, IL-17, IL-6 and IL-1β were also observed in the NG34-stimulated spleen cell supernatants obtained from the CAF01 + NG-34 vaccinated animals (Figures 3A, B, D, F–H). These results could be associated with the role of CAF01 in inducing Th1-cytokines and also with the property of one of the CAF01 compounds (the TDB) which leads to the secretion of IL-1β, IL-6 and TNF-α [35]. Furthermore, the rise in IL-17 levels of the mice from CAF01 + NG-34 (Figure 3F) could be related to the Th17 response that CAF01 promotes through the TLR-independent Syk/Card9-dependent pathway [36] (Figure 2). Other cytokines not from a Th1/Th17 profile were also boosted (IL-10 and IL-6) by CAF01 + NG-34 vaccinated mice (Figures 3D and G). IL-10 induction could be related to the CD4 TFH cells which might promote B cell proliferation, survival, and differentiation into antibody-secreting plasma cells [37–42].

The mice immunized with CAF01 + NG-34 displayed the greatest percentages of B cells population in the GC in the draining lymph nodes (Figure 4A). Other studies have reported CAF01 as an enhancer of GC reaction in comparison to other adjuvants such as AddaVax, aluminum hydroxide and CpG ODN 1826 [43] and recognized that this reaction is primordial for generating plasma and memory B cells [44]. In terms of the percentage of TFH, the highest percentage mean was achieved by the Diluvac Forte + NG-34 but followed immediately by the CAF01 + NG-34 group (Figure 4B). Although not statistically significant, these data are of great relevance since the TFH cells promote long-lasting humoral immunity arising from the GC. CAF01 has been reported to have a more than a year duration inducing CMI responses due to its DDA compound which forms a depot at the injection site [45].

The TDB component of CAF01 is essential for the induction of CMI responses and that it is recognized via the C-type lectin receptor MINCLE (CLEC4E). It is important to note that there may be species-specific differences in recognition and activation of this MINCLE receptor. It is well known that CAF01 is a promising adjuvant in humans, inducing both antibody and CMI responses [16, 34] and also that CAF01 combined with *Chlamydia trachomatis* fusion proteins (Hirep1 and CTH93) induce strong CMI responses in Göttingen minipigs [46]. Nevertheless, whether TDB is also an effective immunostimulator in poultry remains to be assessed and further studies are therefore required to develop the CAF01 + NG-34 as a vaccine for poultry.

As a first step, this study may provide insights into what may be occurring when administering CAF01 + NG-34 to influenza hosts (such as pig and poultry). With regards to the released umbrella of cytokines as well as antibodies induced, CAF01 ought to be a potential adjuvant to be mixed with the conserved NG-34 epitope.

## Data Availability

The data supporting the conclusions of this article is attached within the article and its additional files.

## Abbreviations

AH: aluminium hydroxide
ADCC: antibody-dependent cellular cytotoxicity
ARRIVE: Animal Research Reporting of In Vivo Experiments
BSA: bovine serum albumin
CAF01: cationic adjuvant formulation
CD4/CD8: cluster of differentiation
CMI: cell-mediate immune
CPE: cytopathic effect
DDA: dimethyldioctadecylammonium (DDA)
DMEM: Dulbecco’s Modified Eagle Medium
ELISA: enzyme-linked imunosorbent assay
FBS: fetal bovine serum
GC: germinal centers
HA: hemagglutinin
HI: hemagglutination inhibition
IFN: interferon
Ig: immunoglobulin
IL: interleukin
IVs: influenza viruses
ISM: Informational spectrum methodology
MDCK: Madin-Darby Canine Kidney
OIE: World Organization for Animal Health
PBS: phosphate-buffered saline
RBS: receptor binding site
RPMI: Roswell Park Memorial Institute
QIV: quadrivalent inactivated vaccine
RT: room temperature
SC: subcutaneous
SNT: serum neutralization test
SSI: Statens Serum Institut
TCID_50_: median tissue culture infectious dose
TDB: trehalose 6,6 V-dibehenate
TFH: T follicular helper cells
Th: T helper
TMB: 3, 3′,5,5′-tetramethylbenzidine
TIV: trivalent inactivated vaccine
